# Challenging boundaries: is cross-protection evaluation necessary for African swine fever vaccine development? A case of oral vaccination in wild boar

**DOI:** 10.3389/fimmu.2024.1388812

**Published:** 2024-10-01

**Authors:** Estefanía Cadenas-Fernández, Sandra Barroso-Arévalo, Aleksandra Kosowska, Marta Díaz-Frutos, Carmina Gallardo, Antonio Rodríguez-Bertos, Jaime Bosch, Jose M. Sánchez-Vizcaíno, Jose A. Barasona

**Affiliations:** ^1^ VISAVET Health Surveillance Center, Complutense University of Madrid, Madrid, Spain; ^2^ Department of Animal Health, Faculty of Veterinary, Complutense University of Madrid, Madrid, Spain; ^3^ European Union Reference Laboratory for African Swine Fever (ASF), Centro de Investigación en Sanidad Animal (CISA-INIA/CSIC), Valdeolmos, Spain; ^4^ Department of Internal Medicine and Animal Surgery, Faculty of Veterinary, Complutense University of Madrid, Madrid, Spain

**Keywords:** African swine fever, control disease, cross-protection, virus, vaccine, wild boar

## Abstract

African swine fever (ASF) poses a significant threat to domestic pigs and wild boar (Sus scrofa) populations, with the current epidemiological situation more critical than ever. The disease has spread across five continents, causing devastating losses in the swine industry. Although extensive research efforts are ongoing to develop an effective and safe vaccine, this goal remains difficult to achieve. Among the potential vaccine candidates, live attenuated viruses (LAVs) have emerged as the most promising option due to their ability to provide strong protection against experimental challenges. However, ASF virus (ASFV) is highly diverse, with genetic and phenotypic variations across different isolates, which differ in virulence. This study highlights the limitations of a natural LAV strain (Lv17/WB/Rie1), which showed partial efficacy against a highly virulent and partially heterologous isolate (Arm07; genotype II). However, the LAV's effectiveness was incomplete when tested against a more phylogenetically distant virus (Ken06.Bus; genotype IX). These findings raise concerns about the feasibility of developing a universal vaccine for ASFV in the near future, emphasizing the urgent need to assess the protective scope of LAV candidates across different ASFV isolates to better define their limitations.

## 1 Introduction

The African swine fever virus (ASFV) causes a disease that is highly lethal in naïve populations of domestic pigs and wild boar (*Sus scrofa*) (1). The control of this disease is limited by the lack of an effective vaccine and treatment. Control measures are consequently based on the early detection and rapid implementation of strict health measures that include the slaughter of animals, commercial restrictions, and the systematic closure of national borders throughout the world (2). All of this has an enormous impact on both health and the economy, making this disease the major threat to the global swine industry currently (3). Due to these characteristics, the World Organization for Animal Health (WOAH) considers African swine fever (ASF) as a notifiable disease.

ASF was first discovered in Kenya in 1921, where it was described as a hemorrhagic disease causing high fever in domestic pigs, for which the lethality rate was 100% (4). The first jump to a new continent took place in the 1950s, when it was reported in Portugal (5) and the virus spread rapidly to several European countries, the Caribbean, and South America. After its eradication in the Iberian Peninsula in the 1990s, the disease remained confined to Africa, except for the island of Sardinia (6). However, this situation changed dramatically in 2007, which was when ASF last reentered Europe (Georgia) (7) and subsequently spread uncontrollably through 22 European countries (8). The current epidemiological situation as regards ASF is the most alarming ever. It entered China, the world’s largest producer and consumer of pork, in 2018, and has since spread to the rest of the continent with unprecedented speed and scope - 23 countries affected in only six years (8, 9). ASFV even entered the Caribbean through the Dominican Republic (July 2021); being also declared in Haiti (8).

This alarming epidemiological situation of ASF disease underscores the urgent need for new control tools to limit its spread and contribute to its eradication, particularly the development of an effective and safe vaccine. This urgency is not limited to domestic pigs but also extends to wildlife, where ASF control is especially challenging in this new global ASF scenario. Traditional biosecurity measures, effective in industrialized pig farming, are considerably more complex when applied to wild boar. In fact, routinary strategies have proven inadequate in controlling ASF in this species when the virus is widely spread in sylvatic cycles (10). Early detection of ASF is crucial for effective control in wild boar populations, as evidenced by the successful eradication program in the Czech Republic (11) and Belgium (12). However, in many European countries affected by ASF, wild boar populations remain extensively infected, acting as reservoirs for the disease and posing continuous transmission risks to domestic pigs and neighboring countries (13). The successful Belgian experience with ASF, detailed in Licoppe et al. (2023) (12), further emphasizes this challenge. Despite fewer ASF notifications in Asian wild boars (14), possibly due to sampling bias, the higher prevalence of wild boars compared to Europe (15) suggests a significant reservoir potential for ASF in Asia (16). These insights underscore the necessity of developing an ASF vaccine for wild boars, akin to the pivotal role of vaccination in eradicating classical swine fever in Europe (17).

The search for a vaccine against ASF began more than forty years ago but attaining it has been a great challenge for researchers. The main constraint is the great complexity of the ASFV, which is the only member of its family, *Asfarviridae*, and is considered to be a giant DNA virus that contains more than 150 open-reading frames. Its complexity means that current techniques do not go sufficiently far to attain a full understanding of its structural and functional features (18). The specific antigens that induce a protective immune response are, therefore, unknown. The protective immune response against ASFV is not related to fully neutralizing antibodies and there is evidence that the cellular immune response plays a crucial role (19–21). Moreover, both inactivated vaccines and those based on subunits or recombinants have failed to develop an effective immune response, independently of the adjuvant tested (22–24). This indicates that the viral replication capacity of the vaccine candidate seems relevant for effective protection, signifying that, despite their safety advantages, they do not appear to be the means to attain an ASF vaccine in the short or medium term.

In the realm of controlling ASF, vaccination has emerged as the paramount strategy, particularly in wild boar populations at the European Union level. The urgency for the development of a safe and effective ASF vaccine has never been more apparent. In this sense, live attenuated viruses (LAVs) have consistently demonstrated the highest efficacy against experimental challenges (5, 25, 26). In this context, the WOAH has emphasized the importance of adhering to specific standards in vaccine development, including the need for DIVA (Differentiating Infected from Vaccinated Animals) capabilities, cross-protection against various ASFV strains, and comprehensive safety profiles to mitigate risks like reversion to virulence. Since LAVs stand as the most promising effective vaccine candidates, safety considerations remain a pertinent issue due to the intricate nature of these types of vaccines. Past prototypes, like NH/P68 (27) and OURT88/3 (28), have been linked to chronic forms of ASF, magnifying the inherent risks associated with LAVs, including the potential for reversion to virulence. In response to these concerns and spurred by advances in genetic manipulation, recent years have witnessed the development of live genetically attenuated ASF vaccines through the deliberate deletion of virulence-related genes, aiming to yield safer alternatives (21). On a parallel note, recent research endeavors have concentrated on the creation of modified live vaccines (MLVs) through the targeted deletion of virulence-associated genes on highly virulent ASFV field strains (29). These MLVs exhibit promise by offering complete protection against homologous lethal field strains of ASFV, as demonstrated by the success of ASFV-G-ΔI177L and ASFV-G-ΔMGF—the inaugural commercial ASF vaccines in Vietnam (30, 31). Nonetheless, apprehensions persist over the safety of MLVs, with concerns such as the potential for reversion to virulence or the generation of new variants posing significant challenges (32).

Even though virulence and antigenicity genes are not fully characterized, some genetically attenuated preparations have fortunately proven to be innocuous, producing live vaccines that seem to be safer than those that are naturally attenuated. However, the level of protection induced by these fully innocuous vaccines may be limited, since most of them have been verified only against the parental virulent virus (33–35), which is the same virus from which the virulence-related genes have been deleted. One of the paramount challenges in developing a universally effective vaccine against ASFV lies in its vast phylogenetic and immune-virulence diversity. In this respect, very few studies have tested their protection against isolates other than the parental virus and only one of them has proven to provide protection (36). It would, therefore, seem that safety is not the only issue regarding LAVs, since effectiveness against heterologous isolates also appears to be a challenge, and it is crucial to find a balance between both key points. The ASFV landscape is dotted with a plethora of isolates, each possessing unique genetic and immunogenic characteristics. This immense variability implies that the protective efficacy of a vaccine against one isolate does not guarantee its effectiveness against another. Traditionally, cross-protection among different viral strains is assessed through *in vitro* neutralizing antibody analyses. However, this conventional approach is rendered ineffective in the context of ASFV, given its unique immunological profile. Notably, it is not clear if ASFV does elicit the production of neutralizing antibodies, a phenomenon that significantly complicates the assessment of vaccine-induced immunity (37, 38). The complexity of conducting *in vitro* assays for ASFV is further compounded by its ability to evade standard immunological responses, necessitating a more nuanced approach to vaccine development. The hemadsorption inhibition test has been the primary laboratory assay employed to date (39), yet its reliability remains questionable. Given the increasing evidence suggesting a pivotal role of the cellular immune response in mediating protection against ASFV, and the ambiguous role of antibodies in defending the host against the virus, these *in vitro* assays fall short. Consequently, there is an imperative need for comprehensive *in vivo* studies. These studies not only provide a more holistic understanding of the vaccine’s protective efficacy but also bridge the knowledge gap in our understanding of the complex interplay between ASFV and the host’s immune system.

Despite the important role that wild boar play in the disease, clinical trials for vaccine development have been mainly conducted in domestic pigs. It was not until 2019 that a study of oral wild boar vaccination with an LAV (the Lv17/WB/Rie1 isolate), which proved to be 92% effective against a virulent heterologous isolate, Armenia 2007 (Arm07) was first published (25). To further investigate the safety profile of this vaccine preparation, a study assessing overdose and repeated doses has been undertaken in this species (40). Considering the other concerns regarding ASF vaccines, further studies in terms of cross-protective immunity with this live attenuated vaccine in wild boar are necessary. In our study we have, therefore, tested the scope of the cross-protection induced by Lv17/WB/Rie1 in wild boar. This has been done by challenging animals vaccinated and protected against the virulent virus Arm07, which belongs to the same genotype and clade as the vaccine isolate (genotype II, clade C) (41), with a virulent virus that is more phylogeographically and genetically different, since it belongs to a different genotype and clade (genotype IX, clade A) (41), Kenya 2006 (Ken06.Bus).

## 2 Materials and methods

### 2.1 ASFV isolates

The natural attenuated genotype II ASFV Lv17/WB/Rie1 (clade C) isolate used as an oral vaccine in this study has previously been described and assessed in both domestic pigs and wild boar for immunization purposes (5, 25). The virus was grown in porcine blood monocytes (PBM) for 7 days, after which the culture medium containing extracellular virus was collected and centrifuged at a low speed (2,000 xg, 4°C) in order to remove cellular debris, and then at a high speed (15,000 x g, 4°C) in order to sediment the virus. The viral titer was defined as the amount of virus causing cytopathic effects in 50% of infected cultures (TCID_50_/mL) and was estimated by means of immunoperoxidase staining.

The highly virulent genotype II ASFV Arm07 (clade C) and the genotype IX ASFV Ken06.Bus (clade A) isolates were respectively used as the first and second challenge viruses in order to assess the level of cross-protection of the vaccinated animals. These viruses were propagated in PBM as described previously (42). The viral titer was defined as the amount of virus causing hemadsorption in 50% of infected cultures (HAD_50_/mL).

### 2.2 Animals for experiments

Experiments were performed in the biosafety level 3 facilities at the VISAVET Health Surveillance Centre at the University Complutense of Madrid, Spain. A total of twelve wild boar piglets aged 3-4 months old and obtained from a commercial wild boar farm in Andalusia, Spain, were used in this study. These animals had not been vaccinated against any infectious diseases before the experiment and tested negative for the antibodies of the following infectious diseases: Aujeszky virus, *Mycobacterium bovis*, classical swine fever virus, ASFV, swine vesicular disease virus, *Mycoplasma hyopneumoniae*, porcine reproductive and respiratory syndrome virus (PRRS) and porcine circovirus type 2. The animals were acclimated for 2 weeks before the experiment began.

Animal care, handling, and sampling procedures were conducted in compliance with regional, national and European regulations, and the *in vivo* experimental protocol was given prior approval by the Ethics Committees of the University Complutense of Madrid and the Community of Madrid (reference PROEX 159/19). The protocol included a detailed description of the efforts made to prevent and avoid the animals’ unnecessary suffering, including humane endpoints and euthanasia guidelines, following the Directive 2010/63/UE. All procedures were designed and performed by specifically trained specialists and veterinarians (B, C, and D animal experimentation categories) according to EC Directive 2003/65/EC and Spanish laws RD53/2013. Guidelines for ARRIVE 2.0 for the care and use of laboratory animals were also followed.

### 2.3 Experimental design

**Figure 1:**
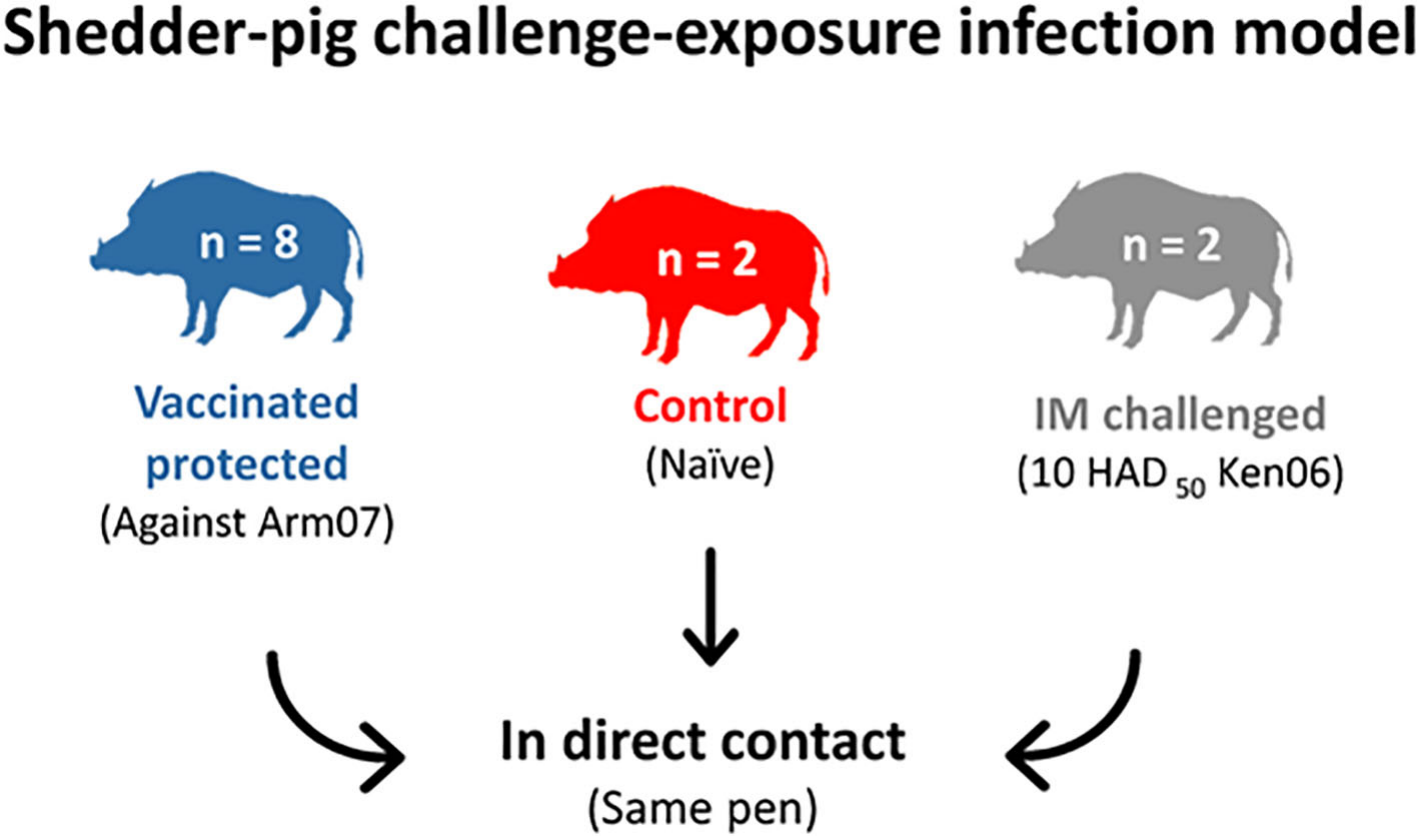
Challenge of the vaccinated wild boar protected with Ken06.Bus following a shedder-pig challenge-exposure infection model.

Eight animals were orally vaccinated with 1 ml of 10^3^ TCID_50_ of ASFV Lv17/WB/Rie1 and were subsequently revaccinated with the same administration dose at 18 days post-vaccination (dpv). The vaccinated animals were then exposed to a first challenge with 10 HAD_50_ of ASFV Arm07 by the intramuscular (IM) route after 42 dpv. Subsequently, 32 days post-IM inoculation with Arm07, all wild boar were exposed to direct contact with two naïve wild boar IM inoculated with 10 HAD_50_ of ASFV Ken06.Bus (IM challenged), following a shedder-pig challenge-exposure infection model (**Figure 1**). Two additional naïve wild boar were placed in-contact with the other wild boar from the onset of the viremia of the IM-challenged animals in order for the former to be used as a control of the challenge. The animals were kept for 28 days post-challenge (dpc), or until they succumbed to the disease, and humane endpoints were consequently established.

Paired EDTA-blood and serum samples were taken twice a week, on 0, 4, 7, 11, 14, 18, 25, and 28 dpc, or on the last day of each animal’s life, for the detection of ASF viral genome and antibodies.

### 2.4 Clinical evaluation

Clinical monitoring was performed daily to examine the animals for any clinical signs of development in order to evaluate the effectiveness of the vaccine prototype against the highly virulent Ken06.Bus isolate. All animals were observed daily throughout the experiment using a 24-h video camera and *in situ* wildlife-specialist veterinarian visits to record their daily clinical signs.

These clinical signs (CS) were expressed in terms of a quantitative CS following the specific guidelines for ASF clinical disease evaluation in wild boar previously described by Cadenas-Fernández et al. (2020) (22). This CS includes rectal temperature, behavior, body condition, skin lesions, ocular/nasal discharge, joint swelling, respiratory, digestive, and neurological symptoms. The only clinical parameter that was not taken daily was rectal temperature in order to minimize the management of the animals, and it was, therefore, measured only twice a week and in animals with any severe symptoms. Fever was defined as a rectal temperature of over 40.0°C.

The key parameters employed to ensure the animals’ welfare were clinical evaluations. The humane endpoint was pre-defined as animals with a CS > 18, and animals with severe clinical signs (level 4) of fever, behavior, body condition, respiratory and digestive signs for more than two consecutive days were also included, following the standards described by Cadenas-Fernández et al. (2020) (22). In addition, any animals undergoing unacceptable suffering without reaching the pre-defined humane endpoint were also euthanized based on veterinarian criteria.

### 2.5 Post-mortem evaluation and tissue sampling

A post-mortem evaluation was performed following the protocol and criteria previously described by Rodriguez-Bertos et al. (2020) (43), which includes specific guidelines for gross findings of ASFV infection in wild boar. During the necropsy, we collected samples from 20 sensitive tissues, eight of which were lymph nodes (renal, mediastinal, retropharyngeal, mesenteric, preescapular, gastrohepatic, inguinal, and mandibular lymph nodes), along with heart, liver, brain, spleen, lung, diaphragm, urinary bladder, kidney, bone marrow, intestine, “meat juice” and synovial membrane. These tissue samples were analyzed in terms of viral genome detection, as described below.

### 2.6 Sample analysis

Serum samples were tested using a commercial ELISA test to detect specific antibodies against ASFV-p72 (INGEZIM PPA Compac K3, Ingenasa-Gold Standard Diagnostics, Madrid, Spain), following the procedure described by the manufacturer. The indirect immunoperoxidase test (IPT) was also used for the analysis of serum. ASFV antibody titers were determined by end-point dilution using IPT, as performed by the European Union Reference Laboratory (5).

The High Pure PCR Template Preparation kit (Roche Diagnostics GmbH, Roche Applied Science, Mannheim, Germany) was used to extract DNA from all tissue homogenates and EDTA-blood samples. The ASF viral genome from blood (hereafter defined as viremia) and tissues was amplified with the Universal Probe Library (UPL) real-time PCR protocol (44). The results were expressed in Cq values (equivalent to cycle threshold, CT), and were considered positive when Cq was < 40.0. Virus isolation was performed using PBM cells as described in the Manual of Diagnostic Tests and Vaccines for Terrestrial Animals (“African Swine Fever,” 2019). The plates were examined for hemadsorption (HAD) over a period of six days and samples were blind passaged three times on PBM. A real-time PCR was conducted after each passaged isolation.

Serum samples were also analyzed in duplicate to simultaneously measure the cytokines IFN alpha, IFN gamma, IL-6, IL-8, IL-10, and TNF alpha at selected time points: day 0 (for vaccinated and control animals); 39 dpv (only vaccinated animals; prechallenge with Arm07); 74 dpv (only vaccinated animals; after challenge with Arm07); and the day of death/sacrifice (for both vaccinated and control animals, after challenge with Ken06.Bus). A fluorescent microbead-based immunoassay was performed using a Porcine Procarta Plex panel (Bendre MedSystems GmbH, Vienna, Austria) specifically configured for the detection of the six analytes, following the manufacturer’s instructions. Fluorescence signals were acquired using a dual-laser BioPlex^®^ 200 instrument (Bio-Rad) and analyzed with Bio-Plex Manager 6.0 software (Bio-Rad).

### 2.7 Data analysis

A descriptive analysis of temperature, CS values, antibodies response, ASFV viremia (Cq values) in blood, and ASFV DNA detection in tissues was performed to estimate average ranges per group and sampling period at 95% confidence intervals. The variation in these parameters among groups and different periods was studied using the Mann-Whitney U test and the Kruskal-Wallis test, respectively. Relationships among continued parametric variables, temperature, CS and Cq values were statistically performed using Spearman’s rank correlations. In addition, the values obtained for each cytokine studied and the sampling time were analyzed using generalized linear models (GzLM) due to non-parametric repeated distributions. These distributions were evaluated by the Kolmogorov-Smirnov test (45). To comparatively assess the relationship of these cytokine levels with the treatment and their time period, considered as explanatory variables, it was decided to use a GzLM with a gamma distribution and logarithmic link (46). All models were subsequently validated to detect overdispersion and to evaluate their residuals and predictors, according to the methods described by Zur et al. (2010) (47). The statistical analysis was carried out using the SPSS 25 statistic program (IBM, Somar, NY, USA) and the 3.5.0 R version software (48). Results were considered statistically significant when p < 0.05.

## 3 Results

### 3.1 Survival rate, viremia, shedding and antibody detection after challenge with Ken06.Bus

**Figure 2:**
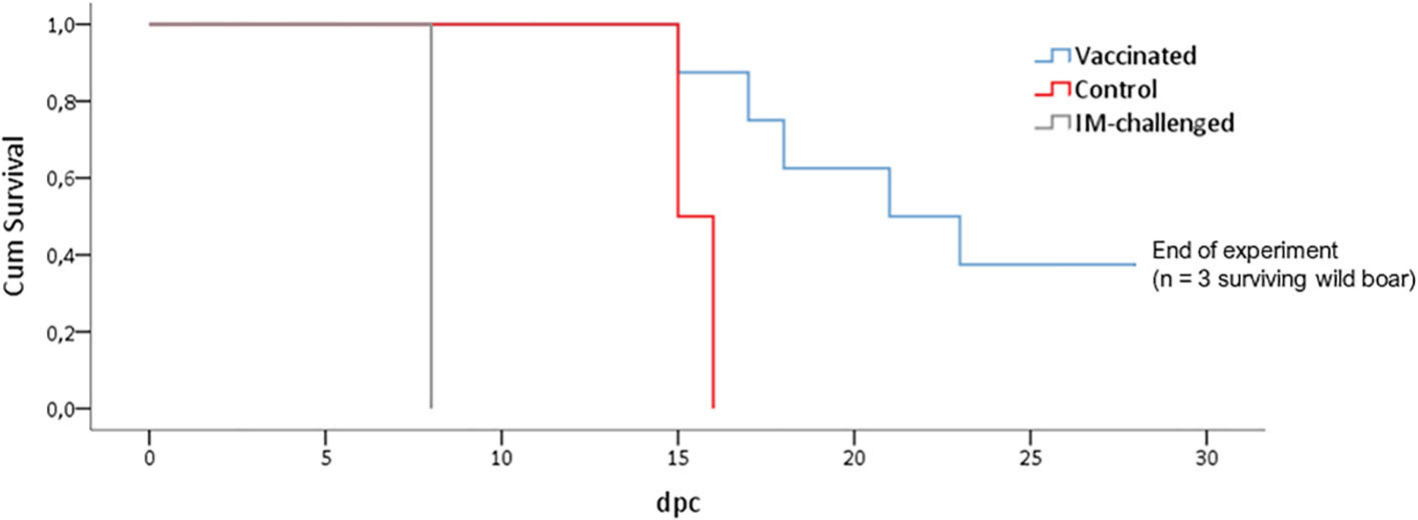
Kaplan-Meier curve showing the evolution of mortality in wild boar orally vaccinated with ASFV Lv17/WB/Rie1 and naïve animals (control) challenged by direct contact with animals IM inoculated with ASFV Ken06.Bus (IM-challenged).

**Figure 3:**
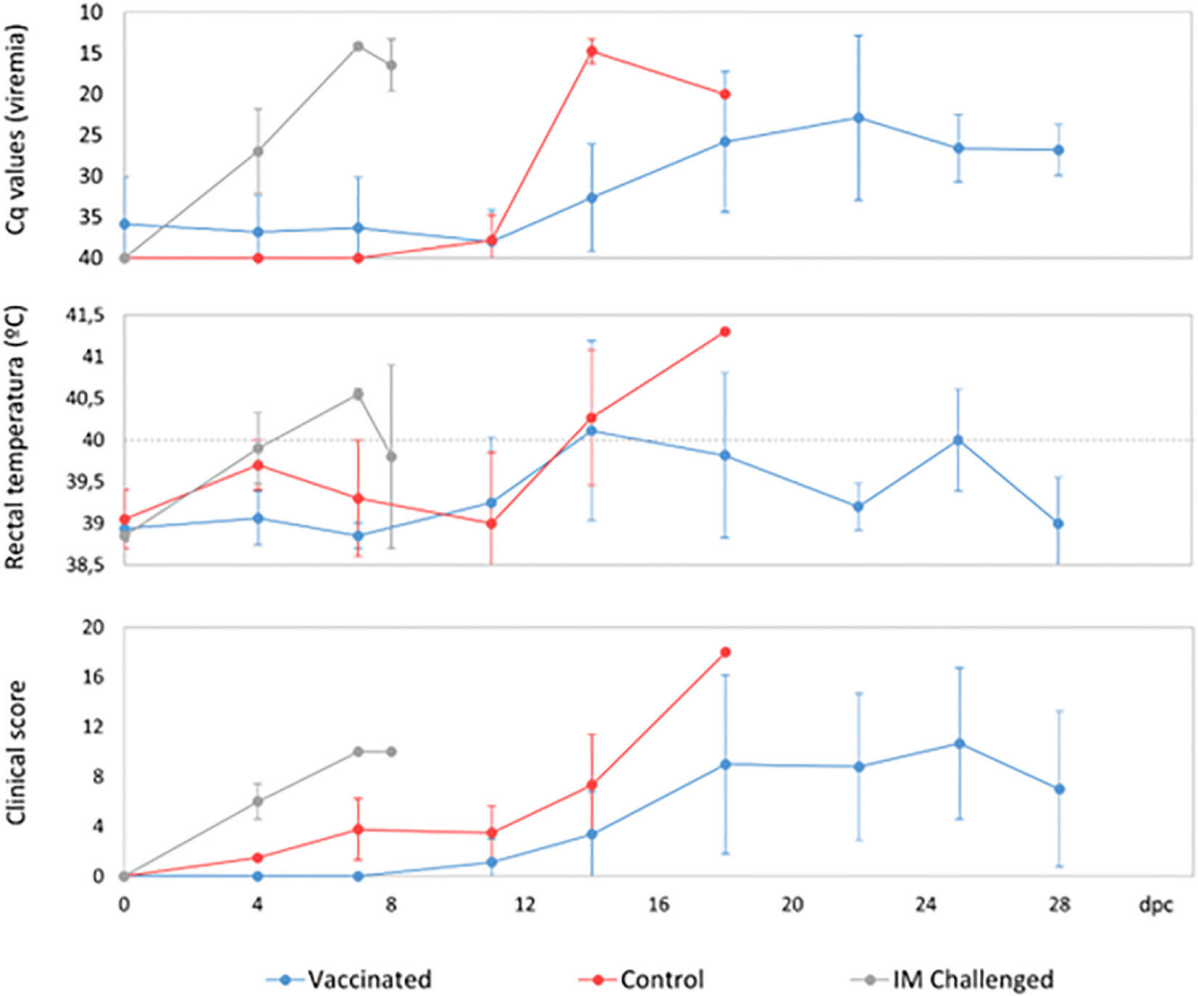
Heat map of Cq values from real-time PCR of blood samples from wild boar. Each row represents an individual animal, and each column represents a time point. The heat map indicates the Cq values for each animal, with color intensity reflecting the magnitude of the values. Animals were either orally vaccinated with ASFV Lv17/WB/Rie1 or were naïve (control), and all were challenged by direct contact with animals intramuscularly inoculated with ASFV Ken06.Bus (IM-challenged). The status of each animal is indicated for better clarity and comparison.

The animals that had been IM-challenged with Ken06.Bus succumbed to the disease at 8 dpc (**Figure 2**). They started to show positive qPCR results of viremia at 4 dpc, which was maintained until the end with a mean Cq value of 19 ± 7 (**Figure 3**). The clinical signs started to appear at 5 ± 1 dpc: first lethargy, a loss of appetite, and fever, with a mean of 40.4 ± 0.2°C (**Figure 3**). The animals then developed localized erythema, slight walking difficulties, and slight dyspnea, after which they got worse and succumbed to the disease with a CS of 10 and 11, respectively (**Figure 3**).

The control animals (**Figure 1**) were likely infected after coming into direct contact with the IM-challenged animals. They developed the disease and succumbed at 16 ± 1 dpc (**Figure 2**). They started to show positive results of viremia at 13 ± 2 dpc, which was also maintained until the end of the experiment, with a mean Cq value of 21 ± 9 (**Figure 3**), without statistically significant differences between them and the IM-challenged animals (Mann-Whitney test, 0.133, p = 0.715). Just after 7 ± 1 dpc, these animals started to show signs of lethargy, but it was not until 14 dpc that they showed other clinical signs such as a loss of appetite, localized slight erythema, slight walking difficulties, moderate dyspnea, and fever, which was observed in only one of the control animals with a mean of 41.3°C (**Figure 3**). The controls subsequently succumbed to the disease with a CS of 11 and 18, respectively (**Figure 3**).

Prior to the challenge with Ken06.Bus, all the vaccinated animals had a positive antibody response to ASFV-p72 according to ELISA and IPT detection. The animals maintained high titers of antibodies throughout the experiment. However, only three vaccinated wild boar survived until the end of the experiment (28 dpc). The remaining five vaccinated animals succumbed to the disease, as did the IM-challenged and control animals. There was a statistically significant difference in survival time between the vaccinated and control animals (Mantel-Cox, x2 = 5.5, 1d.f., p = 0.19). The control animals succumbed to the disease six days before the vaccinated wild boar, which succumbed to the disease at 22 ± 5 dpc (**Figure 2**).

**Figure 4:**
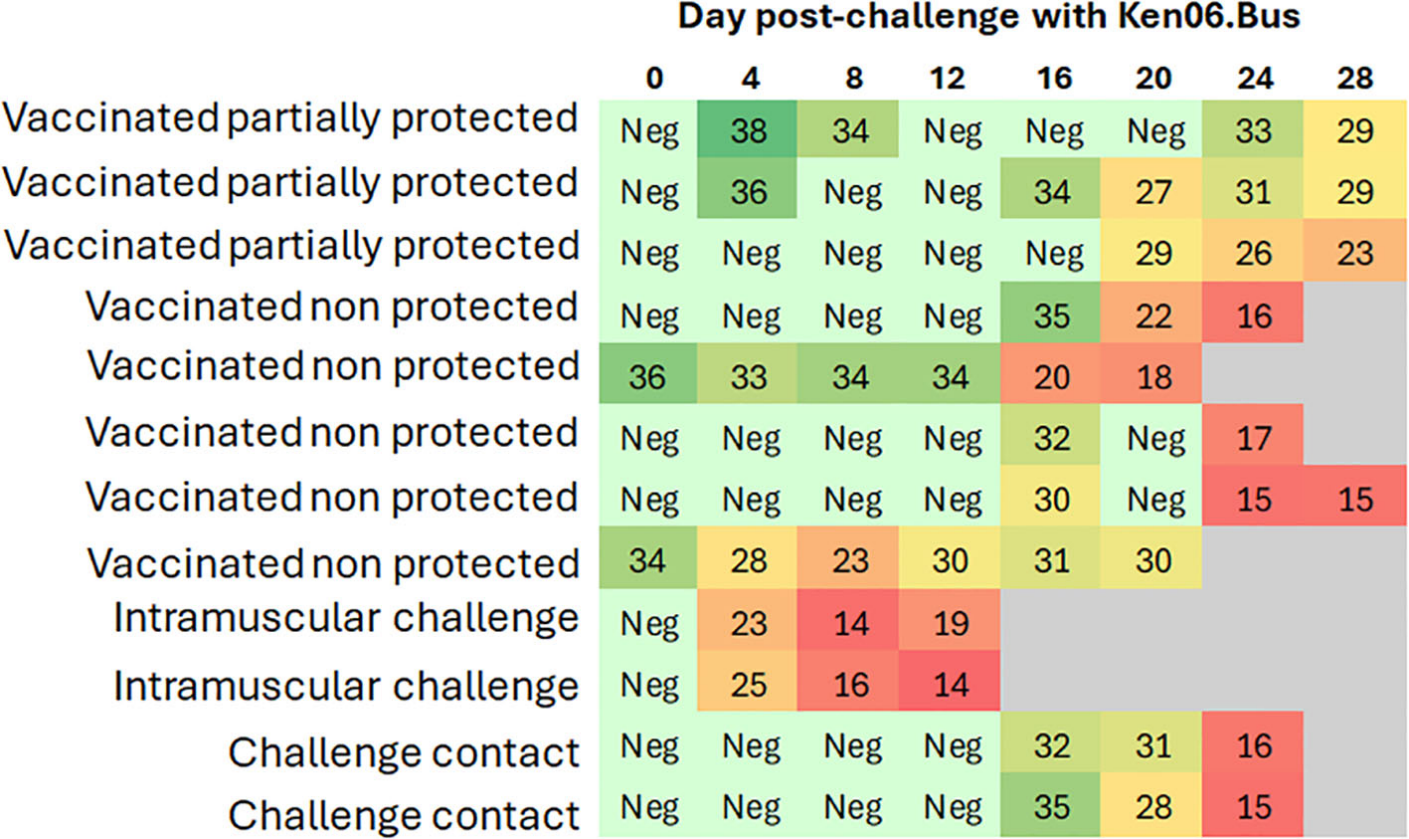
Average clinical scores and rectal temperatures of wild boar orally vaccinated with ASFV Lv17/WB/Rie1 and naïve animals (control) challenged by direct contact with animals IM inoculated with ASFV Ken06.Bus (IM-challenged).

Five vaccinated wild boar maintained earlier viremia, likely of the vaccine virus, attaining positive qPCR results for blood from 0 dpc. Nevertheless, these Cq values had a mean of 32 ± 4, which was significantly higher than that observed in the IM-challenged and control animals (Kruskal-Wallis test, 11.042, p = 0.004; **Figure 3**). Starting at 16 ± 4 dpc, all the vaccinated animals underwent another peak of viremia with a mean Cq value of 25 ± 6 (**Figure 4**), which was more similar to that observed in the IM-challenged and control animals (Kruskal-Wallis test, 4.168, p = 0.124; **Figure 3**). This last increase in viremia was maintained until the end of the experiment.

The vaccinated wild boar started to show clinical signs at 15 ± 4 dpc. The first clinical signs observed in most of these animals were lethargy, loss of appetite, a slight ocular discharge and fever, which were detected in only 5 of the 8 vaccinated wild boar with a mean of 40.7 ± 0.5°C (**Figure 3**). The signs then evolved, and some animals also developed slight to moderate dyspnea, walking difficulties, and neurological signs, and were consequently euthanized with a mean CS of 14 ± 3 (**Figure 3**). Two out of the three vaccinated wild boar that survived until the end of the experiment, developed milder clinical signs than the others and eventually attained a mean CS of 2 and 5, respectively, which was significantly lower than that of the other vaccinated, control and the IM-challenged animals (Kruskal-Wallis test, 4.714, p =0.30).

### 3.2 Cytokine levels in serum

Serum concentrations of various cytokines were analyzed in control animals, non-protected animals against Ken06.Bus, and partially protected animals against Ken06.Bus. The cytokines analyzed include IFN-alpha, IFN-gamma, TNF-alpha, IL-6, IL-8, and IL-10. Measurements were taken at day zero, at 39 days post-vaccination (dpv) following primary and booster vaccinations with the attenuated ASFV Lv17/WB/Rie1, at 74 dpv following the challenge with the virulent Arm07 isolate, and at the final time point after the challenge with the virulent Ken06.Bus isolate.

**Figure 5:**
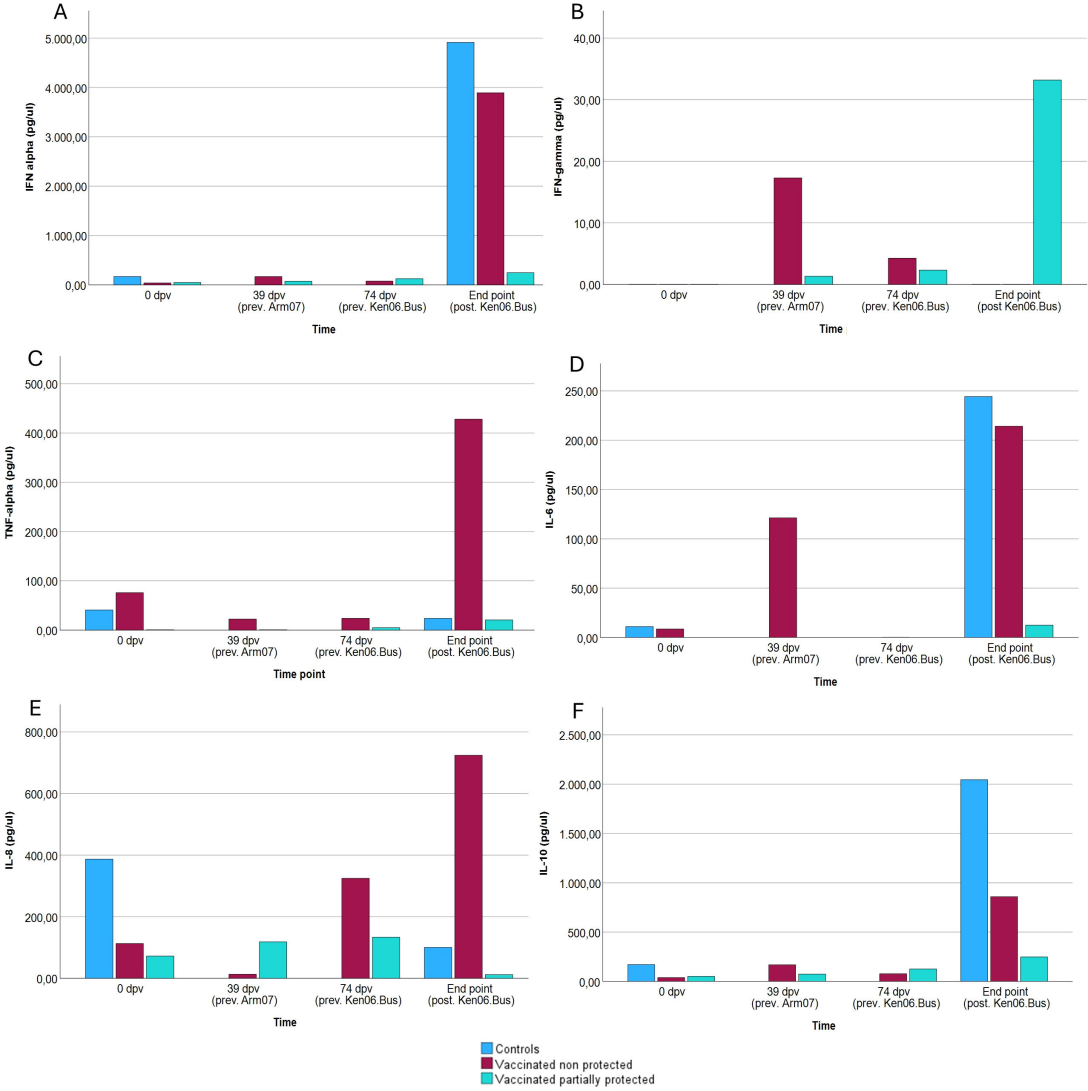
Serum concentration of cytokines in animals over time. Graphs show the levels of **(A)** IFN-alpha, **(B)** IFN-gamma, **(C)** TNF-alpha, **(D)** IL-6, **(E)** IL-8, and **(F)** IL-10 in the serum of control animals (n = 4), non-protected animals against Ken06.Bus (n = 5), and partially protected animals against Ken06.Bus (n = 3). The time points on the X-axis correspond to measurements taken at different post-infection times (prev. Arm07: previous to challenge with Armenia 2007 strain; prev. Ken06.Bus: previous to challenge with Kenya 2006 and after challenge with Arm07; post. Ken06.Bus: after challenge with Ken06.Bus, coinciding with the day of death or sacrifice of each animal).

Statistical analysis was performed using GzLMs, as described in the methodology section. As shown in **Figure 5**, IFN-gamma levels (**Figure 5B**) were significantly higher in the partially protected group compared to the non-protected group following the challenge with Ken06.Bus (β = 374,89; p < 0.05). Regarding IFN-alpha (**Figure 5A**), serum concentration remained more controlled in partially protected animals upon pathogen exposure. Significant differences were found between the partially protected and control groups (p < 0.05), as well as between serum concentrations at the endpoint compared to earlier time points (p < 0.001). Significant differences were observed between the partially protected and non-protected groups (p < 0.001), as well as significant differences at the endpoint compared to pre-challenge time points (p < 0.001). Results showed dysregulated serum levels in control and non-protected animals. Significant differences were observed between the partially protected group and both the control and non-protected groups (p < 0.001), with higher serum IL-6 concentrations in the control and non-protected groups (β = 5.805; p < 0.001). For IL-8 (**Figure 5E**), no statistically significant differences were observed between the three groups (p > 0.05). IL-10 (**Figure 5F**) levels in vaccinated non-protected animals against Ken06.Bus were dysregulated post-challenge, similar to control animals. TNF-alpha (**Figure 5C**) levels remained stable across all groups, including controls, except in vaccinated animals that were not protected against Ken06.Bus. Significant differences were found between the partially protected group and the control group (p < 0.05), with higher serum IL-10 concentrations in both the control and non-protected groups. Additionally, significant differences were found between endpoint concentrations and other study time points (β = 6.249; p < 0.05).

### 3.3 Postmortem studies

All the analyzed tissues from the IM-challenged and controls were positive to qPCR, with mean Cq values of 21 ± 3 and 25 ± 3, respectively, with significant differences (Mann-Whitney test, 5.918, p = 0.001). The tissues analyzed from the vaccinated animals were also all positive, except for those obtained from two animals, which were negative for one and two tissues, respectively. The two animals that survived until the end of the experiment with a lower CS than the rest of the wild boar, also had higher Cq values in their tissues, 31 ± 4 when compared to the other vaccinated animals, 23 ± 3 (Mann-Whitney test, 8.639, p = 0.001), which were more similar to those observed in the IM-challenged and control animals (Kruskal-Wallis test, 0.491, p = 0.623). This means that the two vaccinated animals that survived had a lower viral DNA load in their tissues than did the other vaccinated, control and IM-challenged animals.

The main findings at necropsy were a moderate to severe accumulation of yellowish to reddish fluid in the abdominal cavity (ascites; 58.3%), thorax (hydrothorax; 83.3%) and pericardial sac (hydropericardium; 100%). Pulmonary edema, congestion, and multifocal hemorrhages on the surface of the lung were also observed (**Supplementary Material**). There was congestion and an enlargement of the spleen (splenomegaly; 100%), liver (hepatomegaly; 100%) and lymph nodes (lymphadenomegaly; 100%). Hemorrhages of varying degrees of severity were observed in the lymph nodes, kidney (66.7%) and intestine mucosa (small intestine 16.7% and large intestine 50%).

## 4 Discussion

Substantial progress has been made in recent years as regards the development of ASFV vaccines, but several challenges must still be overcome, such as identifying universal cross-protective immunity or by standardizing assays to evaluate vaccines, thus determining the specific cross-protection capacity of each vaccine. Given the high phylogenetic and immunogenic diversity of ASFV, achieving such universal immunity is a monumental task. The clinical trial described here has assessed a wide scope of the cross-protection induced by the ASFV vaccine candidate Lv17/WB/Rie1 in wild boar after oral administration (25). Previous studies have demonstrated the ability of this prototype to induce safe immunity and effective heterologous protection against the virulent ASFV Arm07 isolate in wild boar (25, 40). However, the complex genetic diversity of ASFV signifies that heterologous protection should be evaluated against more phylogeographical genetically distant isolates in order to confirm its effectiveness as regards cross-protection. Since determinant *in vitro* cross-protection analysis is not possible currently due to the complexity of the virus, clinical trials are required to further evaluate the cross-protective capacity of the different vaccine prototypes. The current study was consequently conducted in order to test whether animals vaccinated and protected against the virulent ASFV Arm07 isolate had immune protection against the virulent ASFV Ken06.Bus isolate; this virus belongs to a different genotype and clade to the vaccine isolate, and it is, therefore, supposed to belong to a different serogroup than Arm07 and Lv17/WB/Rie1, although there is no available information in this regard.

Despite the full protection observed against Arm07 infection, only three of the eight vaccinated animals survived until the end of the experiment (38%) having two of them clinical signs compatible with ASFV infection that were milder than the rest (25%). Moreover, these three animals had lower macroscopic findings and a lower viral DNA load in their tissues than the other vaccinated, control, and IM-challenged animals. All of this indicates that the protective effect of this vaccine candidate decreases considerably when confronted with a virulent virus that is more phylogeographically distant, maintaining only residual protection in some animals. Two potential hypotheses may explain the main outcomes from this study. A first explanation can be that the initial challenge with Arm07 likely acted as a potential booster to the vaccination, further activating the immune cells and enhancing the overall immune response. This booster effect might have played a positive role in the observed cross-protection against Ken06.Bus by keeping the immune system in a heightened state of readiness. On the other hand, it is also possible that the initial challenge with Arm07 led to immune cell exhaustion, thereby weakening the immune system’s ability to effectively counter the subsequent Ken06.Bus challenge (49). The immune cells, having been heavily engaged in combating Arm07, might not have been fully capable of mounting a strong defense against the second, more genetically distant virus. Additionally, our study was designed to reflect real-world scenarios where vaccinated animals could first encounter a less divergent strain before being exposed to a highly divergent one. This sequential exposure model is crucial for understanding the practical efficacy of the vaccine in various epidemiological settings.

Another interesting finding from our study was the fact that the animals remained viremic 28 days after the second challenge, which can be explained by the persistent nature of both virulent and attenuated ASFV strains. Live attenuated vaccines, like Lv17/WB/Rie1, can induce both humoral and cellular immune responses that may not completely clear the virus but rather control and limit its replication. This partial control could account for the persistent low-level viremia observed in our vaccinated animals. For instance, the ASFV-G-ΔI177L vaccine candidate has been shown to persist in inoculated pigs for at least 49 days post-vaccination, with viral genomes detected in the tonsil and spleen (31, 50). Extending the duration of follow-up in future studies would provide a more comprehensive understanding of the vaccine’s efficacy over time, as ASFV can persist for extended periods, and longer observation is crucial for assessing complete viral clearance and the establishment of sterile immunity.

Furthermore, the cytokine results provide additional insights into the immune response dynamics in partially protected versus non-protected animals. Significantly higher levels of IFN-gamma in the partially protected group indicate its crucial role in coordinating effective immune responses against virulent strains, both innate and adaptive. IFN-gamma plays a key role in activating macrophages, enhancing antigen presentation, and promoting the cytotoxic activity of NK cells and CD8+ T cells, as shown in various studies. This cytokine is essential for both early defense against infections and the development of long-term adaptive immunity. The controlled IFN-alpha levels in these animals also suggest a well-regulated antiviral response, essential for limiting viral replication and spread. IFN-alpha is known to induce an antiviral state in cells and modulate the immune response to prevent excessive inflammation, which is critical for controlling viral infections without causing significant tissue damage. Conversely, the excessive TNF-alpha release in vaccinated animals that were not protected against Ken06.Bus suggests an overactive inflammatory response, possibly contributing to pathology rather than protection. This dysregulation underscores the complexity of the immune response, where a balance between pro-inflammatory and anti-inflammatory signals is vital for effective protection. IL-6 levels, which were higher in control and non-protected animals, further illustrate this point, as its dual role can both promote and suppress inflammation, potentially aiding in viral dissemination when not properly regulated. Its dysregulation can facilitate viral dissemination and worsen disease outcomes. The lack of significant differences in IL-8 levels among the groups indicates that this cytokine may not play a major role in differential protection in this context. In contrast, the elevated IL-10 levels in non-protected animals highlight its role in modulating immune responses, potentially suppressing effective antiviral activity and facilitating viral persistence. The distinct cytokine dynamics observed in partially protected animals suggest that achieving a balanced immune response is key to effective protection, providing valuable insights for future vaccine development.

From the few prototypes of ASF vaccines that have been studied, this lack of cross-protection has been suggested, even among isolates that are phylogenetically closer to each other. This is the case of the vaccine prototype based on the naturally attenuated virus OURT88/3 (genotype I, clade C), which provides effective protection against the homologous virus OURT88/1, but has shown a lack of protection against the heterologous virus MOZ98/1 (genotype VIII, clade B) (51), and also against the heterologous virus Benin97/1 (genotype I, clade C), which belongs to the same genotype (36). Although the naturally attenuated virus NH/P68 (genotype I, clade C) has provided cross-protection against the heterologous challenge with Arm07 (genotype II, clade C), it lost its protective capacity when confronted with the same heterologous challenge when this NH/P68 virus was genetically modified (27). This raises questions about the stability and predictability of immunity induced by genetically modified strains and supports the idea that genetically attenuated vaccine prototypes are more related to cross-protection failures.

In this line, it is necessary to highlight that very few genetically attenuated viruses have, to date, achieved a protective immune response to heterologous virulent ASFV. This is the case of the Georgia 2007/1 virus with the deletion of the I177L gene (ASFV-G-ΔI177L), which has been shown to provide protection against virulent Vietnamese ASFV field isolates (52). The BA71 virus with the deletion of the viral protein CD2 (BA71ΔCD2) has also been shown to provide heterologous protection, which is dose-dependent against the Georgia 2007/1 virus (genotype II, clade C) (53), and provides 33% protection against the same virus used in the current study, the Ken06.Bus isolate (54). It is crucial to note that while these vaccines show promise, the variability in protection levels suggests that multiple factors, beyond just genetics, play a role in determining vaccine efficacy. This underscores the importance of understanding the underlying mechanisms of immunity and how they interact with different ASFV strains.

Several studies have pointed out the role of ASFV serotype-specific proteins in cross-protection. For instance, Burmakina et al. (2016) (55) demonstrate that ASFV proteins CD2v and C-type lectin are significant for protection against homologous ASFV infection, suggesting their potential as key protective antigens in vaccine design. Indeed, most of the vaccine prototypes based on genetic attenuation by gene deletion have been tested only against their parental viruses (33, 34, 56), that is, the base virus from which the genetic modification is obtained. Considering the remaining concerns regarding vaccine development, further studies in terms of cross-protective immunity with LAVs in pig and wild boar are necessary.

ASFV has great genetic variation among isolates. Descriptions of 24 different genotypes based on the p72 capsid protein gene have been provided to date (57). However, this classification involves only epidemiologic and geographic data (58). While p72-based genotyping of ASFV offers insights, it does not convey antigenic information. As previously noted, animals that are protected against specific isolates may still lack cross-protection against other isolates within the same genotype (59), and this is, therefore, of limited value as regards predicting the cross-protection efficacy of vaccines. Given the importance of cellular immunity in ASFV protection, relying solely on these genotypic classifications may not provide a complete picture of potential vaccine efficacy. Furthermore, there is a serologic classification of ASFV based on the serological typing by haemadsorption inhibition (HAI), and eight different serogroups have been identified to date (59, 60). Protective immunity against ASFV currently appears to be serogroup-specific, but new research should be carried out to determine its robustness and the genetic and antigenic bounds of cross-protective immunity. Two critical points for the development of vaccines against ASFV can be attained from all of the above. The first is that the evaluation of cross-protective immunity is essential since there is great genetic and antigenic variation among ASFV isolates and it is necessary to establish the scope of heterogeneous protection. The other is that the cross-protection efficacy of ASF vaccines cannot currently be predicted with the available methodologies and standards, signifying that *in vivo* clinical trials should be carried out.

Based on the clinical vaccination trials carried out to date, and despite advances in effective protection against specific ASFV isolates, heterologous protection remains understudied, and outcomes are mostly negative. This emphasizes the need for a more holistic approach to vaccine development that considers the genetic and immunological intricacies of ASFV. The complexity and limitations of cross-protection studies indicate the unpredictable nature of vaccine efficacy, since different results are observed even for the same type of vaccine, highlighting the need for *in vivo* clinical trials. All of this suggests that achieving a universal vaccine for ASF, providing protection against all virulent isolates, may be challenging in the short term. Consequently, vaccine effectiveness studies must prioritize evaluating heterologous protection and establishing the specific bounds for each vaccine prototype.

In conclusion, this study aptly reflects the multifaceted challenges and complexities associated with achieving cross-protection in ASF vaccines, emphasizing the need for thorough evaluation and consideration of both genetic and immunological factors in vaccine development.

## Data Availability

The original contributions presented in the study are included in the article/**Supplementary Material**. Further inquiries can be directed to the corresponding author.
