# Supplementary material for: Challenging boundaries: is cross-protection evaluation necessary for African swine fever vaccine development? A case of oral vaccination in wild boar
Source: Front Immunol. 2024 Oct 1;15:1388812. doi: 10.3389/fimmu.2024.1388812 (PMC11473374; doi:10.3389/fimmu.2024.1388812)
Supplement: Supplementary file 1 [file DataSheet1.docx]

Supplementary Material

Challenging boundaries: Is cross-protection evaluation necessary for African swine fever vaccine development? A case of oral vaccination in wild boar

**Estefanía Cadenas-Fernández^1,2^, Sandra Barroso-Arévalo^1,2*,^ Aleksandra Kosowska^1,2^, Carmina Gallardo^4^, Antonio Rodríguez-Bertos^1,3^, Jaime Bosch^1,2^, Jose M. Sánchez-Vizcaíno^1,2^, Jose A. Barasona^1,2^**

^1^ VISAVET Health Surveillance Center, Complutense University of Madrid, 28040 Madrid, Spain

^2^ Department of Animal Health, Faculty of Veterinary, Complutense University of Madrid, 28040 Madrid, Spain

^3^ Department of Internal Medicine and Animal Surgery, Faculty of Veterinary, Complutense University of Madrid, 28040 Madrid, Spain

^4^ European Union Reference Laboratory for ASF; Centro de Investigación en Sanidad Animal (CISA-INIA/CSIC), Valdeolmos, 28130 Madrid, Spain

*** Correspondence:**Sandra Barroso-Arévalo
sandrabarroso@ucm.es

# Supplementary Figures

**
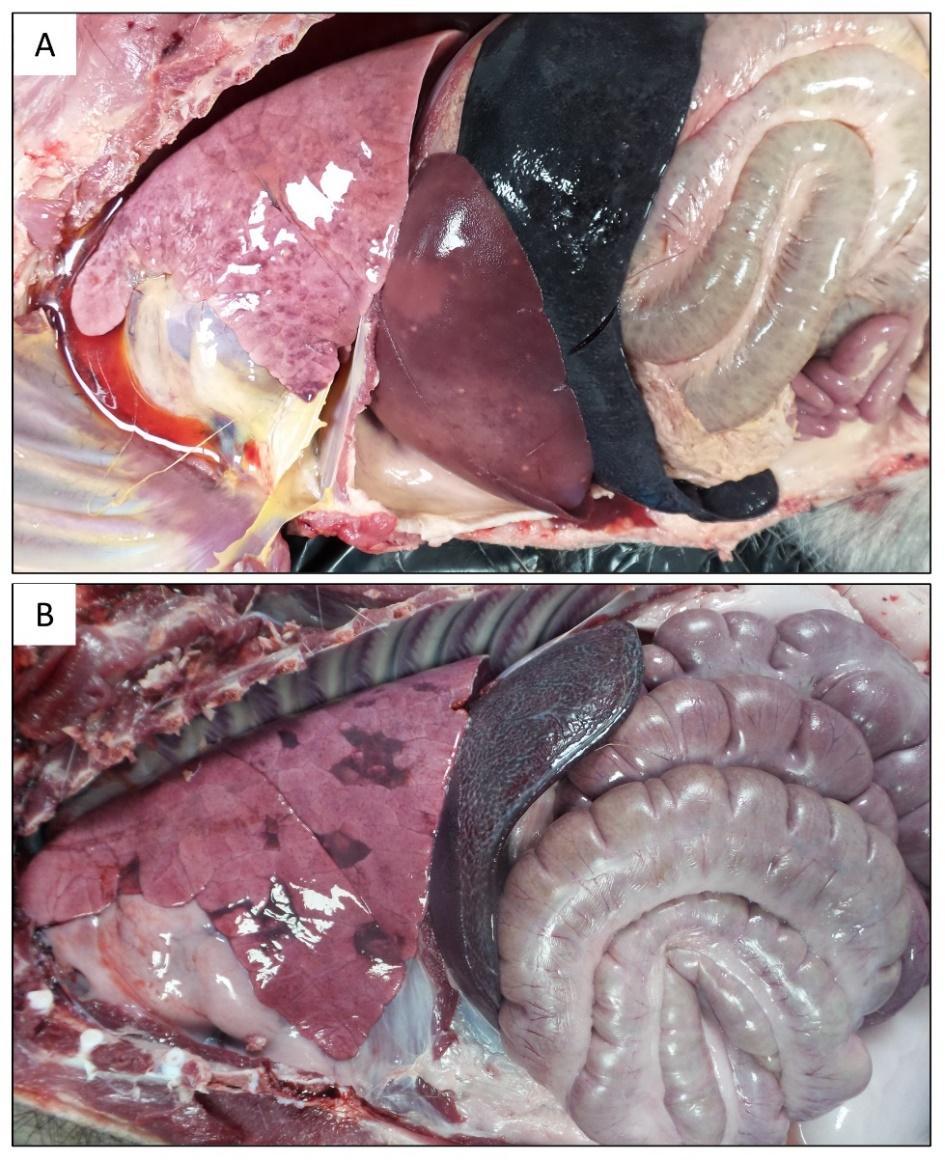
**

**Supplementary Figure 1.** View of thoracic and abdominal cavities from naïve wild boar (A, control) and orally immunized wild boar (B, vaccinated) both subsequently challenged by contact with Ken06.Bus. Hydrothorax, pulmonary edema and congestion, splenomegaly and hepatomegaly are evident in (A).
